# Effect of reducing dietary crude protein level on growth performance, blood profiles, nutrient digestibility, carcass traits, and odor emissions in growing-finishing pigs

**DOI:** 10.5713/ab.23.0155

**Published:** 2023-06-26

**Authors:** Aaron Niyonsaba, Xing Hao Jin, Yoo Yong Kim

**Affiliations:** 1 Department of Agricultural Biotechnology and Research Institute of Agriculture and Life Science, Seoul National University, Seoul 08826, Korea

**Keywords:** Blood Profiles, Growing-finishing Pigs, Growth Performance, Low-protein Diet, Odor Emissions

## Abstract

**Objective:**

This study was conducted to evaluate the effect of a low-protein diet on growth performance, carcass traits, nutrient digestibility, blood profiles, and odor emissions in growing-finishing pigs.

**Methods:**

A total of 126 crossbred pigs ([Yorkshire×Landrace]×Duroc) with an average body weight (BW) of 38.56±0.53 kg were used for a 14-week feeding trial. Experimental pigs were allotted to one of 6 treatments in 3 replicates of 7 pigs per pen in a randomized complete block design. Pigs were fed each treatment diet with different levels of crude protein (CP). Phase 1 (early growing): 14%, 15%, 16%, 17%, 18%, 19%; phase 2 (late growing): 13%, 14%, 15%, 16%, 17%, 18%; phase 3 (early finishing): 12%, 13%, 14%, 15%, 16%, 17%; phase 4 (late finishing): 11%, 12%, 13%, 14%, 15%, 16%. All experimental diets in each phase were contained the same concentration of lysine (Lys), methionine (Met), threonine (Thr), and tryptophan (Trp).

**Results:**

Over the entire experimental period, there was no significant difference in BW, average daily feed intake, and gain-to-feed ratio among all treatments (p>0.05), but a quadratic effect (p = 0.04) was observed in average daily gain (ADG) during the late finishing phase with higher ADG in Group D. Blood urea nitrogen concentration linearly increased with an increase in dietary CP levels (p<0.01). Regarding nutrient digestibility, excreted nitrogen in urine and feces and nitrogen retention linearly increased as the CP level increased (p<0.01). A linear effect was observed with increasing CP levels in amines, ammonia, and hydrogen sulfide in odor emissions (p<0.01). No significant effects were observed in the measurements of carcass traits and meat characteristics (p>0.05).

**Conclusion:**

In phase feeding, reducing the CP level to 14% in early-growing pigs, 13% in late-growing pigs, 12% in early-finishing pigs, and 11% in late-finishing pigs is recommended.

## INTRODUCTION

In swine production, dietary protein is the most important nutrient because it not only provides essential amino acids (EAAs) but also greatly affects the total cost of production. To meet the lysine requirement of pigs, the quantity of soybean meal was increased during diet formulation, which ultimately resulted in high crude protein (CP) levels [1]. When dietary protein exceeds the requirements of pigs, the surplus is excreted either in urine or in feces as nitrogenous waste products such as ammonia and amines. This can affect production economy and the environment by increasing the waste of protein ingredients, feed cost, and nitrogen emissions. To mitigate the consequences caused by high CP levels in the diet, the low-protein (LP) diet supplemented with crystalline amino acids was regarded as an effective strategy [2]. However, it is almost impossible to supplement all the EAAs required by pigs to ensure the efficiency of nitrogen deposition, and supplementation with limiting amino acids (LAAs) (Lys, Met, Trp, and Thr) or even fewer AAs has been adopted as a common strategy in pig production [3].

As stated above, reducing the CP level has been a promising solution to mitigate the consequences caused by high CP levels in the swine diet. Adopting the approach of LP diet usage can save protein ingredients, reduce nitrogen excretion, lower feed costs, and mitigate the risk of gut disorders [4,5]. In this regard, dietary CP reduction within 3% of the NRC [6] and supplementation with LAA can result in similar growth performance in growing-finishing pigs [7]. However, there have been discrepancies regarding whether CP can be reduced by 4% or more. It was reported that reducing dietary CP by 4% and supplementing with LAA showed no effect on the growth performance of pigs from growing to the finishing period [8,9]. Conversely, a decrease in average daily gain (ADG) and growth performance in 20 to 50 kg pigs was reported when dietary protein levels were reduced by 4% and 5%, respectively, with LAA supplementation [10,11]. Most likely, this was due to the imbalance of EAAs in LP diets [3]. It has been controversial whether pigs fed LP diets have fatter carcasses than those fed higher protein diets [8,12]. In addition, approximately 3 decades ago, Jørgensen et al [13] reported higher ileal digestibility of fat with increasing dietary CP, but Adams and Jensen [14] found a lower digestibility of crude fat with increasing dietary CP. Moreover, ammonia was prioritized to estimate the degree of odor emissions caused by dietary CP, but hydrogen sulfide was given little attention in the measurement of odor emissions, although it can cause harmful consequences such as the death of animals and humans [15] and regional acid rain [16]. Thus, more studies are needed to examine whether there can be a further reduction in dietary CP levels without causing any detrimental effects.

Therefore, this study was conducted to investigate the effects of reducing dietary CP levels on growth performance, carcass traits, nutrient digestibility, blood profiles, and odor emissions in growing-finishing pigs.

## MATERIALS AND METHODS

### Experimental animals and management

All experimental procedures involving animals were conducted in accordance with the Animal Experimental Guidelines provided by the Seoul National University Institutional Animal Care and Use Committee (SNUIACUC; SNU-210811-6).

A total of 126 crossbred pigs ([Yorkshire×Landrace]×Duroc) with an average body weight (BW) of 38.56±0.53 kg were used for a 14-week feeding trial. Pigs were reared at the Seoul National University experimental farm in the facilities for growing-finishing pigs (2.60×2.84 m). Feed and water were provided *ad libitum* during the entire experimental period by a 6-hole stainless feeder and two nipples installed in each pen. Based on the collected data of BW and feed intake at the end of each phase, the ADG, average daily feed intake (ADFI), and gain-to-feed (G:F) ratio were calculated step by step in each phase. Feed supply to all the treatments was recorded each day, and waste feed left in the feeder was recorded at the end of each phase.

### Experimental design and diet

The pigs were allotted to one of six treatments considering sex and initial BW in 3 replications with three male pigs and four female pigs per pen in a randomized complete block design. Experimental diets were formulated for 4 phases: phase 1 (early growing phase) was weeks 0 to 4; phase 2 (late growing phase) was weeks 5 to 7; phase 3 (early finishing phase) was weeks 8 to 11; and phase 4 (late finishing phase) was weeks 12 to 14. Pigs were fed each treatment diet with different levels of CP (Phase 1: 14%, 15%, 16%, 17%, 18%, 19%; Phase 2: 13%, 14%, 15%, 16%, 17%, 18%; Phase 3: 12%, 13%, 14%, 15%, 16%, 17%; Phase 4: 11%, 12%, 13%, 14%, 15%, 16%). All nutrients in the experimental diets except CP met or exceeded the nutrient requirements for 25 to 125 kg pigs according to the NRC [17]. The formula and chemical compositions of the experimental diets are provided in Tables 1, 2, 3, and 4.

### Blood sampling and analysis

In each treatment, six randomly selected pigs of near-average BW were used to collect blood samples on the initial day and at the end of each phase. Blood samples were taken from the jugular vein to measure blood urea nitrogen (BUN), total protein, glucose, and creatinine during the growing and finishing phases. Blood samples were collected in a disposable culture tube and centrifuged for 15 min at 3,000 rpm and 4°C (Eppendorf centrifuge 5810R, Hamburg, Germany). The serum was carefully transferred to 1.5-mL plastic tubes and stored at −20°C for later analysis. BUN (kinetic UV assay; Roche, Mannheim, Germany) and glucose (enzymatic kinetic assay, Roche, Germany) concentrations were analyzed using a blood analyzer. The creatinine and total protein concentrations were measured by kinetic colorimetry assay using a blood analyzer (Modular Analytics, PE; Roche, Germany).

### Nutrient digestibility and odor emissions

A digestibility trial was conducted two times in a completely randomized design with two replicates to evaluate nutrient digestibility and nitrogen retention. An experimental diet of the early growing phase was provided to each treatment animal. A total of 12 crossbred growing barrows, averaging 41.35± 1.45 kg BW, were individually allotted to each treatment and housed in metabolic crates. A total collection method was utilized for apparent digestibility. After 5 days of the adaptation period, pigs were subjected to 5 days of collection, and 0.4% ferric oxide and chromium oxide were used as initial and end markers, respectively. The diets were fed to pigs twice a day at 08:00 and 17:00, at a rate of 2.0 times the maintenance requirement for metabolizable energy, and the pigs had *ad libitum* access to water. Excreta and urine were collected daily and stored at −20°C for later analysis. At the end of the trial, the excreta were dried (68°C, 72 h) in an air-drying oven and ground (5-mm screen, Wiley mill) for chemical analysis.

For odor gas estimation, at the end of the digestibility trial, 150 g of fresh feces and 100 g of urine were mixed in a 4.2 L plastic box. A mixture of fecal and urine was fermented at a room temperature of 28°C for 48 hours. The odor-causing gases (ammonia [NH_3_], amines [R·NH_2_], and hydrogen sulfide [H_2_S]) were analyzed with a gas detector (GV-110S; Gastec Corp, Kanagawa, Japan) and tube, namely, NH_3_ detector tube No. 3L, R·NH_2_ detector tube No. 180L, and H_2_S detector tube No. 4LL.

### Carcass traits and meat characteristics

At the end of the experiment, four pigs from each treatment group were selected and slaughtered at an average of 114.22 kg±1.42 for carcass analysis. Pork samples were collected near the 10th rib on the right side of the carcass. Because of the chilling procedure, 30 minutes after slaughter was regarded as the initial time. The longissimus muscle’s pH and color were measured at 0, 3, 6, 9, 12, and 24 hours. The pork samples were always stored in the freezer (4°C). The pH was measured using a pH meter (Model 720; Thermo Orion, Fullerton, CA, USA), and pork color was measured by Commission Internationale de l’Eclairage (CIE) color L*, a*, and b* values using a CR300 (Minolta Camera Co., Osaka, Japan). Chemical analysis of pork samples was conducted using the AOAC method [18]. The water-holding capacity (WHC) of pork was measured by the centrifuge method. Longissimus muscles were ground and sampled in a filter tube, heated in a water bath at 80°C for 20 min and centrifuged for 10 min at 2,688×g at 10°C (5810R; Eppendorf, Germany).

Lipid oxidation was determined by calculating 2-thiobarbituric acid reactive substances (TBARS) values. Briefly, each sample (3 g) and 9 mL of distilled water were homogenized (Ika) with 50 mL of Butylated hydroxytoluene (BHT) (7.2%) for 30 s (1,130×g). The homogenate (1 mL) was transferred to a 15-mL test tube and then mixed with 2 mL thiobarbituric acid (20 mM)/trichloroacetic acid (15%) solution. The tubes were then heated for 30 min in a water bath (90°C), cooled, and centrifuged at 2,688×g at 10°C (5810R; Eppendorf, Germany). The absorbance of the supernatant was measured at 532 nm using a spectrophotometer. To calculate cooking loss, longissimus muscles were packed in a polyethylene bag that was then heated in a water bath until the core temperature reached 72°C and weighed before and after cooking. After heating, samples were cored (0.5 inches in diameter) parallel to the muscle fiber, and the cores were used to measure shear force using a salter (Warner Barzler Shear, Norwood, MA, USA). The cooking loss, shear force, TBARS, and WHC of pork were analyzed by the Animal Origin Food Science laboratory at Seoul National University.

### Statistical analyses

All collected data were statistically analyzed by least squares mean comparisons using a general linear model procedure of SAS (SAS Institute Inc., Cary, NC, USA). Orthogonal polynomial contrasts were performed to analyze the linear or quadratic effects of reducing dietary CP levels in the diet. Each pen was considered an experimental unit for measuring growth performance. An individual pig was used as a unit for data on nutrient digestibility, blood profile, odor emissions, and carcass traits. Statistical differences were considered highly significant at p<0.01, significant at p<0.05. Treatment effects were considered to have a tendency when the probability was between 0.05 and 0.10.

## RESULTS AND DISCUSSION

### Growth performance

The effect of different dietary CP levels on growth performance is demonstrated in Table 5. During the entire experimental period, there were no significant differences in BW, ADFI, and G:F ratio among all treatments (p>0.05). However, a quadratic effect (p = 0.04) was observed in ADG during the late finishing phase, with a higher value in pigs of treatment Group D.

The effect of a low CP-level diet on growth performance in growing-finishing pigs has been a topic of debate among researchers for many years. It is commonly believed that reducing dietary CP within 3% of the NRC [6] and supplementing with limiting AA cannot affect the growth performance in growing-finishing pigs [7]. In addition, previous studies show that a reduction of 4% in CP level had no significant effect on growth performance in pigs from growing to finishing when supplemented with crystalline Lys, Trp, Thr, and Met [8–9]. However, Yi et al [10] reported that reducing dietary protein by 4% decreased the ADG in 20 to 50 kg pigs. Moreover, Roux et al [19] reported a significant decrease in growth performance after reducing the CP level by 4.8% along with limiting AA supplementation in growing pigs. In the present study, reducing the CP level in the diet to 14% in 25 to 50 kg pigs, 13% in 50 to 75 kg pigs, 12% in 75 to 100 kg pigs, and 11% in 100 to 125 kg pigs did not result in a significant difference in growth performance compared to high-protein diets. Therefore, our data strongly supported that in phase feeding, reducing CP levels to the above-stated range did not affect the growth performance of growing-finishing pigs. A quadratic effect was found in ADG within the treatment of Group D. It was speculated that since the pigs from this group had relatively lower BW values compared to other groups at the start of the late finishing phase, these pigs had still the space for nutrient deposition which subsequently led to their accelerated growth and higher ADG than other groups in the late finishing phase.

### Blood profiles

The results of blood profiles during the feeding trial are presented in Table 6. Total protein, glucose, and creatinine had no significant differences during the whole experimental period (p>0.05), but BUN concentration linearly increased as dietary CP levels increased during all phases (p<0.01).

Animals excrete ammonia by converting it to urea in the liver, and from there, it is released into the blood and then travels to the kidneys, where it is excreted in urine [20]. Thus, BUN is an indicator of whether protein intake is excessive in the diet and amino acid utilization in pigs [21]. Our BUN data agreed with previous studies that observed a reduction in BUN concentration by lowering CP in the diet [22,23]. Bergsjo et al [24] reported that insufficient protein intake can decrease the levels of blood total protein. A higher serum total protein level is an indicator of improvement in the pig’s protein status [25]. Therefore, according to the current study, the reduction in dietary CP level did not have any detrimental effect on the protein status of pigs. Van der Schoor et al [26] that reported if there was a deficit of intestinal fuels such as glutamate, glutamine, and asparagine in LP diets, EAAs can act as intestinal cell energy fuel. Blood creatinine has a direct relationship with body muscle content [27]. In our data, no significant differences were observed in serum glucose and creatinine. Thus, we suggest that reducing the CP level to the range we used in our study could not affect glucose-AA metabolism and muscle production.

### Nutrient digestibility and odor emissions

The effect of different dietary CP levels on nutrient digestibility is shown in Table 7. In this trial, excreted nitrogen in urine and feces and nitrogen retention linearly increased as the CP level increased (p<0.01). These data were supported by our results of BUN where BUN increased as the CP level increased, which ultimately might increase the amount of excreted N in urine. This was consistent with the report by O’Connell et al [28], who showed that pigs offered diets containing 220 g CP/kg excreted significantly more urinary and fecal nitrogen and showed higher retained nitrogen than those offered diets containing 160 g CP/kg. N retention can be influenced by multiple factors such as AA imbalances, different digestible AA levels, or different efficiencies of AA utilization among diets. According to our findings, we suggest that an increase in dietary CP level increased the balance and variety of amino acids available for digestion which eventually increased N retention [29]. The difference in dietary CP did not affect the digestibility of dry matter (DM), protein, and ash (p>0.05), but fat digestibility tended to linearly decrease as the CP level increased (p = 0.07). Jørgensen et al [13] reported a higher ileal digestibility of fat and saturated fatty acids with increasing dietary CP in growing pigs. However, Adams and Jensen [14] stated that a lower digestibility of crude fat is observed with increasing dietary CP. It was speculated that the reason for fat digestibility decrease was that free fatty acids could bind with undigested proteins to form micelles that were not able to be digested. Previous studies reported that lowering CP levels reduced nutrient digestibility in growing and weaning pigs [30]. Lower digestive enzyme activity in the gastrointestinal tract of nursery and growing pigs fed LP diets was likely the cause of this depression [4]. It should be noted that there was a lack of significant difference in our CP, ash, and DM digestibility results because the pigs we used in the trial were old enough to have strong digestive enzyme activity.

The effect of different dietary CP levels on odor emissions is illustrated in Table 8. A linear effect was observed with increasing CP levels in amines, ammonia, and hydrogen sulfide (p<0.01).

Currently, odor emissions are a problem of concern in commercial pig production because of their contribution to environmental pollution. Anaerobic fermentation of undigested proteins in animal manure is the main cause of the production of odorants; hence, the reduction of dietary CP could be a good solution for decreasing odor emissions [31]. The current data were in line with the findings of many previous authors who reported the reduction of nitrogenous gas in LP diets [32,33]. Ammonia emission from feces and urine can be reduced by 8% to 10% when 10 g/kg of CP is decreased from the diet [31]. In this study, an average reduction of 8.5% in ammonia was observed for every 1% reduction in dietary CP. Although H_2_S has received less attention than ammonia in past odor emission studies, it has deleterious effects on organisms and the environment. These effects include the deaths of animals and human beings exposed to H_2_S [15] and the risk of acid rain when it is emitted into the atmosphere [16]. One of the sources of hydrogen sulfide in pig facilities is the fermentation products of sulfur-containing AA originating from dietary protein [16]. Some studies on the effect of CP levels on hydrogen sulfide emissions showed no significant effects [34,35]. Interestingly, a linear relationship between CP level and hydrogen sulfide emissions was found in the present study. It is beyond the scope of our analysis to explain in detail the cause for this correlation, but we postulate that the combination of supplemented methionine and the proportion of sulfur-containing amino acids, which increased by raising the CP levels, could be the reason. Therefore, we recommend that more studies are needed to reveal a connection between hydrogen sulfide emission and dietary CP or supplemented crystalline amino acids.

### Carcass traits

The effect of dietary CP levels on carcass characteristics is presented in Table 9. There were no significant differences in carcass moisture, CP, crude fat, or crude ash in all treatments (p>0.05). Similarly, the difference in CP levels showed no significant effect (p>0.05) on the physiochemical properties of cooking loss, shear force, TBARS, and water holding capacity.

Kerr et al [8] reported that reducing CP levels by supplementing synthetic amino acids can increase carcass fat deposition, and more available net energy for fat deposition in the LP diet could be the main reason. Interestingly, no significant effect was found in crude fat or other measured proximate factors among lower and higher CP-level diets in our study. Stewart et al [36] also showed that no significant effects were found on moisture, CP, crude fat, or crude ash in pork resulting from tested pig diets (growing diets, 17.83% to 20.30%; finishing diets, 12.44% to 15.21%). In addition, no effect on carcass characteristics was found after reducing the CP level by supplementing limiting AA in growing-finishing pigs [3,30]. Moreover, Fang et al [37] and Hong et al [38] reported that decreasing the CP level in the diet did not affect meat-proximate factors (18% to 11.2% and 18% to 13.2%, respectively). In addition to the proximate analysis, variations in diet CP level had no detrimental effects on cooking loss, shear force, TBARS, or WHC. The result in TBARS is in line with that of Fang et al [37], who found no significant difference in TBARS with reducing dietary protein levels in the diet. Leaner pork increases shear force and cooking loss and decreases WHC, and cooking loss can be a measure of WHC since lower cooking loss results in higher WHC [38]. In addition, a reduction in dietary protein in the diet of pigs affects intramuscular fat and marbling of the longissimus muscle [8]. In the current study, it is notable that there was no significant change in crude fat content in longissimus muscle.

### Meat characteristics

The results of the effect of dietary CP levels on the pH and meat color of the longissimus muscle are shown in Table 10 and 11. In this study, there were no significant differences in pH and meat color (L*, a*, and b*) at any measured points.

The pH influences the color of pork, and a lower pH decreases the color, while a higher ultimate pH leads to a darker color. In the present study, variation in dietary CP levels did not show any significant effect on either pH or meat color, and no correlation was observed between them. These results agree with those of Zhu et al [39], who found that LP diets supplemented with amino acids have no significant effects on longissimus muscle pH or meat color L*, a*, or b* values of the CIE system. In addition, Prandini et al [7] reported that decreasing the CP level in the diet did not affect meat color. Li et al [40] also reported that finishing pigs’ diets with different CP levels (10% vs 14%) had no significant effects on the meat color of the longissimus dorsi muscle, pH at 45 min, and pH at 24 hours.

## CONCLUSION

In this study, reducing dietary CP levels had no detrimental effect on the parameters of growth performance, carcass traits, and nutrient digestibility, but BUN and excreted N linearly increased with increasing dietary CP levels. According to present results, the CP level to 14% in early-growing pigs, 13% in late-growing pigs, 12% in early-finishing pigs, and 11% in late-finishing pigs is recommended.

## Figures and Tables

**Table 1:** Formula and chemical composition of the experimental diet in early-growing pigs

Items	Protein levels					

	CP14	CP15	CP16	CP17	CP18	CP19
Ingredients (%)						
Ground corn	74.84	71.83	68.83	65.82	62.81	59.81
Soybean meal	16.01	19.18	22.35	25.54	28.71	31.88
Wheat bran	4.00	4.00	4.00	4.00	4.00	4.00
Tallow	1.40	1.51	1.61	1.72	1.83	1.93
L-Lysine-HCl, 50%	0.76	0.61	0.46	0.30	0.15	0.00
DL-met, 99%	0.07	0.06	0.04	0.03	0.01	0.00
L-threonine, 98.5%	0.23	0.18	0.14	0.09	0.05	0.00
L-tryptophan, 99%	0.08	0.06	0.05	0.03	0.02	0.00
Di-calcium phosphate	1.40	1.33	1.26	1.20	1.13	1.06
Limestone	0.71	0.73	0.75	0.78	0.80	0.82
Vit. Mix^1)^	0.10	0.10	0.10	0.10	0.10	0.10
Min. Mix^2)^	0.10	0.10	0.10	0.10	0.10	0.10
Salt	0.30	0.30	0.30	0.30	0.30	0.30
Total	100.00	100.00	100.00	100.00	100.00	100.00
Chemical composition^3)^						
Metabolizable energy (Kcal/kg)	3,300.00	3,300.00	3,300.00	3,300.00	3,300.00	3,300.00
Crude protein (%)	14.00	15.00	16.00	17.00	18.00	19.00
Calcium (%)	0.66	0.66	0.66	0.66	0.66	0.66
Total phosphorous (%)	0.56	0.56	0.56	0.56	0.56	0.56
SID AA (%)^4)^						
Lysine	0.98	0.98	0.98	0.98	0.98	0.98
Methionine	0.31	0.31	0.31	0.31	0.31	0.31
Threonine	0.67	0.67	0.67	0.67	0.67	0.67
Tryptophan	0.20	0.20	0.20	0.20	0.20	0.20

CP, crude protein; SID AA, standardized ileal digestible amino acids.

1) Provided per kg of diet: vitamins per kg of complete diet: vitamin A, 8,000 IU; vitamin D_3_, 1,800 IU; vitamin E, 40 IU; vitamin K_3_, 4 mg; thiamine, 2.00 mg; riboflavin, 7.0 mg; pantothenic acid, 20 mg; niacin, 50 mg; pyridoxine, 3 mg, d-biotin, 0.2 mg; folic acid, 1 mg; vitamin B_12_, 0.03 mg.

2) Provided per kg of diet: mineral per kg of complete diet: Se, 0.3 mg; I, 0.3 mg; Mn, 49 mg; Cu, 288 mg; Fe, 150 mg; Zn, 85 mg; Co, 2 mg.

3) Calculated value.

4) SID AA: Lysine met, but methionine, threonine, and tryptophan exceeded NRC (2012) AA requirements by (Met, 0.03; Thr, 0.08; Trp, 0.03).

**Table 2:** Formula and chemical composition of the experimental diet in late-growing pigs

Items	Protein levels					

	CP13	CP14	CP15	CP16	CP17	CP18
Ingredients (%)						
Ground corn	77.17	74.34	71.51	68.68	65.85	63.03
Soybean meal	14.13	17.13	20.13	23.12	26.12	29.12
Wheat bran	4.00	4.00	4.00	4.00	4.00	4.00
Tallow	1.16	1.27	1.38	1.48	1.59	1.69
L-Lysine-HCl, 50%	0.76	0.61	0.46	0.30	0.15	0.00
DL-met, 99%	0.07	0.06	0.04	0.03	0.01	0.00
L-threonine, 98.5%	0.23	0.18	0.14	0.09	0.05	0.00
L-tryptophan, 99%	0.08	0.06	0.05	0.03	0.02	0.00
Di-calcium phosphate	1.27	1.20	1.14	1.07	1.01	0.94
Limestone	0.63	0.65	0.67	0.68	0.70	0.72
Vit. Mix^1)^	0.10	0.10	0.10	0.10	0.10	0.10
Min. Mix^2)^	0.10	0.10	0.10	0.10	0.10	0.10
Salt	0.30	0.30	0.30	0.30	0.30	0.30
Total	100.00	100.00	100.00	100.00	100.00	100.00
Chemical composition^3)^						
Metabolizable energy (Kcal/kg)	3,300.00	3,300.00	3,300.00	3,300.00	3,300.00	3,300.00
Crude protein (%)	13.00	14.00	15.00	16.00	17.00	18.00
Calcium (%)	0.59	0.59	0.59	0.59	0.59	0.59
Total phosphorous (%)	0.52	0.52	0.52	0.52	0.52	0.52
SID AA (%)^4)^						
Lysine	0.85	0.85	0.85	0.85	0.85	0.85
Methionine	0.28	0.28	0.28	0.28	0.28	0.28
Threonine	0.69	0.69	0.69	0.69	0.69	0.69
Tryptophan	0.21	0.21	0.21	0.21	0.21	0.21

CP, crude protein; SID AA, standardized ileal digestible amino acids.

1) Provided per kg of diet: vitamins per kg of complete diet: vitamin A, 8,000 IU; vitamin D_3_, 1,800 IU; vitamin E, 40 IU; vitamin K_3_, 4 mg; thiamine, 2.00 mg; riboflavin, 7.0 mg; pantothenic acid, 20 mg; niacin, 50 mg; pyridoxine, 3 mg, d-biotin, 0.2 mg; folic acid, 1 mg; vitamin B_12_, 0.03 mg.

2) Provided per kg of diet: mineral per kg of complete diet: Se, 0.3 mg; I, 0.3 mg; Mn, 49 mg; Cu, 288 mg; Fe, 150 mg; Zn, 85 mg; Co, 2 mg.

3) Calculated value.

4) SID AA: Lysine met, but methionine, threonine, and tryptophan exceeded NRC (2012) AA requirements by (Met, 0.04; Thr, 0.17; Trp, 0.06).

**Table 3:** Formula and chemical composition of the experimental diet in early-finishing pigs

Items	Protein levels					

	CP12	CP13	CP14	CP15	CP16	CP17
Ingredients (%)						
Ground corn	80.49	77.66	74.83	71.99	69.16	66.33
Soybean meal	11.35	14.35	17.35	20.34	23.34	26.34
Wheat bran	4.00	4.00	4.00	4.00	4.00	4.00
Tallow	0.89	1.00	1.11	1.21	1.32	1.43
L-Lysine-HCl, 50%	0.76	0.61	0.46	0.30	0.15	0.00
DL-met, 99%	0.07	0.06	0.04	0.03	0.01	0.00
L-threonine, 98.5%	0.23	0.18	0.14	0.09	0.05	0.00
L-tryptophan, 99%	0.08	0.06	0.05	0.03	0.02	0.00
Di-calcium phosphate	1.01	0.94	0.88	0.81	0.75	0.68
Limestone	0.62	0.64	0.66	0.68	0.70	0.72
Vit. Mix^1)^	0.10	0.10	0.10	0.10	0.10	0.10
Min. Mix^2)^	0.10	0.10	0.10	0.10	0.10	0.10
Salt	0.30	0.30	0.30	0.30	0.30	0.30
Total	100.00	100.00	100.00	100.00	100.00	100.00
Chemical composition^3)^						
Metabolizable energy (Kcal/kg)	3,300.00	3,300.00	3,300.00	3,300.00	3,300.00	3,300.00
Crude protein (%)	12.00	13.00	14.00	15.00	16.00	17.00
Calcium (%)	0.52	0.52	0.52	0.52	0.52	0.52
Total phosphorous (%)	0.47	0.47	0.47	0.47	0.47	0.47
SID AA (%)^4)^						
Lysine	0.73	0.73	0.73	0.73	0.73	0.73
Methionine	0.27	0.27	0.27	0.27	0.27	0.27
Threonine	0.65	0.65	0.65	0.65	0.65	0.65
Tryptophan	0.20	0.20	0.20	0.20	0.20	0.20

CP, crude protein; SID AA, standardized ileal digestible amino acids.

1) Provided per kg of diet: vitamins per kg of complete diet: vitamin A, 8,000 IU; vitamin D_3_, 1,800 IU; vitamin E, 40 IU; vitamin K_3_, 4 mg; thiamine, 2.00 mg; riboflavin, 7.0 mg; pantothenic acid, 20 mg; niacin, 50 mg; pyridoxine, 3 mg, d-biotin, 0.2 mg; folic acid, 1 mg; vitamin B_12_, 0.03 mg.

2) Provided per kg of diet: mineral per kg of complete diet: Se, 0.3 mg; I, 0.3 mg; Mn, 49 mg; Cu, 288 mg; Fe, 150 mg; Zn, 85 mg; Co, 2 mg.

3) Calculated value.

4) SID AA: Lysine met, but methionine, threonine, and tryptophan exceeded NRC (2012) AA requirements by (Met, 0.06; Thr, 0.19; Trp, 0.07).

**Table 4:** Formula and chemical composition of the experimental diet in late-finishing pigs

Items	Protein levels					

	CP11	CP12	CP13	CP14	CP15	CP16
Ingredients (%)						
Ground corn	83.68	80.85	78.02	75.19	72.36	69.53
Soybean meal	8.60	11.60	14.60	17.59	20.59	23.59
Wheat bran	4.00	4.00	4.00	4.00	4.00	4.00
Tallow	0.66	0.76	0.87	0.98	1.09	1.19
L-Lysine-HCl, 50%	0.76	0.61	0.46	0.30	0.15	0.00
DL-met, 99%	0.07	0.06	0.04	0.03	0.01	0.00
L-threonine, 98.5%	0.23	0.18	0.14	0.09	0.05	0.00
L-tryptophan, 99%	0.08	0.06	0.05	0.03	0.02	0.00
Di-calcium phosphate	0.84	0.77	0.70	0.64	0.57	0.50
Limestone	0.58	0.60	0.62	0.65	0.67	0.69
Vit. Mix^1)^	0.10	0.10	0.10	0.10	0.10	0.10
Min. Mix^2)^	0.10	0.10	0.10	0.10	0.10	0.10
Salt	0.30	0.30	0.30	0.30	0.30	0.30
Total	100.00	100.00	100.00	100.00	100.00	100.00
Chemical composition^3)^						
Metabolizable energy (Kcal/kg)	3,300.00	3,300.00	3,300.00	3,300.00	3,300.00	3,300.00
Crude protein (%)	11.00	12.00	13.00	14.00	15.00	16.00
Calcium (%)	0.46	0.46	0.46	0.46	0.46	0.46
Total phosphorous (%)	0.43	0.43	0.43	0.43	0.43	0.43
SID AA (%)^4)^						
Lysine	0.61	0.61	0.61	0.61	0.61	0.61
Methionine	0.26	0.26	0.26	0.26	0.26	0.26
Threonine	0.61	0.61	0.61	0.61	0.61	0.61
Tryptophan	0.18	0.18	0.18	0.18	0.18	0.18

CP, crude protein; SID AA, standardized ileal digestible amino acids.

1) Provided per kg of diet: vitamins per kg of complete diet: vitamin A, 8,000 IU; vitamin D_3_, 1,800 IU; vitamin E, 40 IU; vitamin K_3_, 4 mg; thiamine, 2.00 mg; riboflavin, 7.0 mg; pantothenic acid, 20 mg; niacin, 50 mg; pyridoxine, 3 mg, d-biotin, 0.2 mg; folic acid, 1 mg; vitamin B_12_, 0.03 mg.

2) Provided per kg of diet: mineral per kg of complete diet: Se, 0.3 mg; I, 0.3 mg; Mn, 49 mg; Cu, 288 mg; Fe, 150 mg; Zn, 85 mg; Co, 2 mg.

3) Calculated value.

4) SID AA: Lysine met, but methionine, threonine, and tryptophan exceeded NRC (2012) AA requirements by (Met, 0.08; Thr, 0.21; Trp, 0.07).

**Table 5:** The effects of different dietary crude protein levels on growth performance in growing-finishing pigs

Criteria	Treatment^1)^						SEM	p-value	
	
	A	B	C	D	E	F		Linear	Quadratic
Body weight (kg)									
Initial	38.57	38.61	38.54	38.45	38.59	38.60	-	-	-
4 wk	59.26	56.72	58.39	56.26	59.53	56.92	2.800	0.94	0.94
7 wk	74.98	72.50	71.86	70.10	74.53	71.92	3.001	0.86	0.78
11 wk	102.27	99.84	97.12	94.08	96.78	94.04	4.012	0.56	0.34
14 wk	118.43	115.79	114.92	114.45	115.69	113.72	2.883	0.73	0.88
ADG (g)									
0 to 4 wk	738.95	646.60	708.84	638.10	747.96	664.29	41.675	0.89	0.83
5 to 7 wk	780.99	728.53	641.50	658.73	714.29	714.06	24.557	0.51	0.18
8 to 11 wk	952.66	991.13	907.94	856.46	973.30	780.98	20.219	0.12	0.26
12 to 14 wk	781.41	760.00	848.11	944.49	706.73	843.86	20.136	0.40	0.04
0 to 14 wk	813.43	781.57	776.60	774.44	778.45	750.80	13.851	0.33	0.88
ADFI (kg)									
0 to 4 wk	1.87	1.77	1.81	1.63	1.75	1.68	0.091	0.60	0.84
5 to 7 wk	2.65	2.49	2.61	2.32	2.61	2.51	0.102	0.23	0.96
8 to 11 wk	3.09	2.81	3.25	2.64	3.02	2.82	0.210	0.15	0.20
12 to 14 wk	2.89	2.86	3.20	3.25	2.90	3.19	0.041	0.16	0.25
0 to 14 wk	2.66	2.48	2.72	2.46	2.57	2.48	0.058	0.49	0.95
G:F ratio									
0 to 4 wk	0.39	0.36	0.39	0.38	0.42	0.39	0.006	0.19	0.85
5 to 7 wk	0.29	0.29	0.25	0.29	0.27	0.28	0.014	0.43	0.35
8 to 11 wk	0.30	0.31	0.28	0.30	0.32	0.27	0.018	0.25	0.87
12 to 14 wk	0.31	0.26	0.24	0.27	0.23	0.26	0.005	0.32	0.57
0 to 14 wk	0.31	0.31	0.29	0.31	0.31	0.31	0.004	0.57	0.41

SEM, standard error of the mean; ADG, average daily gain; ADFI, average daily feed intake; G:F ratio, gain-to-feed ratio.

1) A, 14% to 11%; B, 15% to 12%; C, 16% to 13%; D, 17% to 14%; E, 18% to 15%; F, 19% to 16%.

(Reduced CP level by 1% for every phase in each treatment group throughout all 4 phases)

**Table 6:** The effects of different dietary crude protein levels on blood profiles in growing-finishing pigs

Items	Treatment^1)^						SEM	p-value	
	
	A	B	C	D	E	F		Linear	Quadratic
Blood urea nitrogen (mg/dL)									
4 wk	5.73	6.15	10.53	11.35	15.48	17.20	0.973	<0.01	0.57
7 wk	6.90	7.40	11.30	11.90	12.17	15.29	0.826	<0.01	0.64
11 wk	7.00	9.40	11.90	14.40	14.42	16.85	0.803	<0.01	0.35
14 wk	8.93	9.10	12.25	14.27	15.17	16.27	0.705	<0.01	0.65
Total protein (g/dL)									
4 wk	6.65	6.40	6.75	6.85	6.65	6.72	0.056	0.28	0.64
7 wk	6.92	6.77	6.85	6.70	6.70	6.85	0.063	0.60	0.44
11 wk	6.85	6.82	7.07	6.70	6.87	7.10	0.053	0.34	0.41
14 wk	6.95	6.77	7.12	7.02	7.02	7.12	0.053	0.18	0.98
Glucose (mg/dL)									
4 wk	87.25	86.00	87.00	93.00	94.50	87.00	1.034	0.10	0.15
7 wk	79.75	77.32	78.00	82.50	80.00	81.50	0.891	0.24	0.69
11 wk	88.77	90.00	87.32	87.25	92.00	90.00	0.880	0.52	0.51
14 wk	82.00	84.50	82.00	82.50	78.00	83.75	0.692	0.42	0.55
Creatinine (mg/dL)									
4 wk	1.04	1.23	1.12	1.00	1.19	1.01	0.025	0.38	0.14
7 wk	1.29	1.20	1.27	1.31	1.35	1.16	0.034	0.80	0.47
11 wk	1.11	1.24	1.21	1.18	1.23	1.17	0.023	0.60	0.26
14 wk	1.35	1.35	1.22	1.29	1.28	1.29	0.025	0.40	0.40

SEM, standard error of the mean.

1) A, 14% to 11%; B, 15 to 12%; C, 16% to 13%; D, 17% to 14%; E, 18% to 15%; F, 19% to 16%.

(Reduced CP level by 1% for every phase in each treatment group throughout all 4 phases)

**Table 7:** The effects of different dietary crude protein levels on nutrient digestibility in growing pigs

Criteria	Treatment^1)^						SEM	p-value	
	
	CP14	CP15	CP16	CP17	CP18	CP19		Linear	Quadratic
Nutrient digestibility (%)									
Dry matter	89.61	89.96	89.36	90.61	90.69	89.75	0.174	0.22	0.31
Crude protein	87.62	87.23	86.43	88.53	88.87	86.72	0.271	0.39	0.57
Crude ash	63.60	62.08	61.50	58.41	59.23	61.53	0.685	0.12	0.11
Crude fat	74.73	73.77	74.32	73.92	73.49	70.09	0.642	0.07	0.27
N-retention (g/d)									
N-intake	19.94	21.70	22.86	23.44	24.89	27.06	0.471	-	-
N-feces	2.47	2.77	3.10	2.68	3.26	3.59	0.094	<0.01	0.41
N-urine	9.53	10.25	10.70	11.70	12.00	12.80	0.296	<0.01	0.95
N-retention^2)^	7.94	8.67	9.06	9.04	9.63	10.67	0.261	<0.01	0.63

CP, crude protein; SEM, Standard error of the mean; N, nitrogen.

1) The diet for phase 1 was used in the nutrient digestibility trial with two replicates for each treatment.

2) N retention = N intake (g)−Fecal N (g)−Urinary N (g).

**Table 8:** The effects of different dietary crude protein levels on odor emissions in growing pigs

Criteria	Treatment^1)^						SEM	p-value	
	
	CP 14	CP 15	CP 16	CP 17	CP 18	CP 19		Linear	Quadratic
Odor emissions (ppm)									
Amines	24.66	30.33	37.00	45.00	56.66	66.33	3.638	<0.01	0.12
Ammonia	10.55	12.80	15.33	18.37	20.88	23.39	1.125	<0.01	0.88
Hydrogen sulfide	1.39	1.43	1.57	1.87	1.97	2.00	0.06	<0.01	0.36

CP, crude protein; SEM, standard error of the mean; ppm, parts per million.

1) The diet for phase 1 was used in the gas emissions trial.

**Table 9:** The effects of different dietary crude protein levels on carcass characteristics

Criteria	Treatment^1)^						SEM	p-value	
	
	A	B	C	D	E	F		Linear	Quadratic
Proximate analysis									
Moisture	68.74	74.29	68.31	71.14	66.91	69.57	0.711	0.21	0.57
Crude protein	25.09	23.91	24.72	23.82	25.28	24.87	0.004	0.80	0.50
Crude fat	6.91	5.63	6.82	5.89	6.81	6.61	0.159	0.70	0.18
Crude ash	0.71	0.70	0.57	0.50	0.73	0.39	0.060	0.19	0.86
Physiochemical properties									
Cooking loss	32.44	31.99	36.7	35.94	31.68	31.33	2.448	0.90	0.39
Shear force	31.47	34.72	32.35	27.46	33.14	30.01	0.842	0.30	0.98
WHC	62.38	67.60	63.43	65.49	62.44	67.80	0.978	0.50	0.81
TBARS	0.143	0.143	0.152	0.130	0.126	0.130	0.004	0.12	0.74

SEM, standard error of the mean; WHC, water-holding capacity; TBARS, 2-thiobarbituric acid reactive substances.

1) A, 14% to 11%; B, 15% to 12%; C, 16% to 13%; D, 17% to 14%; E, 18% to 15%; F, 19% to 16%.

(Reduced CP level by 1% for every phase in each treatment group throughout all 4 phases)

**Table 10:** The effects of different dietary crude protein levels on the pH of longissimus muscle

Criteria	Treatment^1)^						SEM	p-value	
	
	A	B	C	D	E	F		Linear	Quadratic
pH									
0 h	5.70	5.92	5.77	5.62	5.81	5.58	0.0798	0.58	0.52
3 h	5.60	5.75	5.56	5.56	5.56	5.56	0.0430	0.36	1.00
6 h	5.57	5.71	5.58	5.54	5.54	5.58	0.0396	0.96	0.52
9 h	5.58	5.69	5.57	5.57	5.56	5.56	0.0328	0.85	0.34
12 h	5.56	5.69	5.56	5.58	5.58	5.57	0.0362	0.73	0.57
24 h	5.57	5.94	5.57	5.57	5.58	5.57	0.0525	0.36	0.48

SEM, standard error of the mean.

1) A, 14% to 11%; B, 15% to 12%, C, 16% to 13%; D, 17% to 14%; E, 18% to 15%; F, 19% to 16%.

(Reduced CP level by 1% for every phase in each treatment group throughout all 4 phases)

**Table 11:** The effects of different dietary crude protein levels on meat color

Criteria	Treatment^1)^						SEM	p-value	
	
	A	B	C	D	E	F		Linear	Quadratic
CIE value, L*									
0 h	34.51	33.58	39.94	35.32	35.98	38.44	1.759	0.57	0.89
3 h	35.63	30.30	38.89	41.38	40.41	36.73	1.042	0.22	0.15
6 h	44.54	41.29	42.64	42.50	44.79	41.93	1.219	0.92	0.85
9 h	39.00	39.54	37.71	39.10	45.00	40.60	1.446	0.42	0.85
12 h	34.44	38.23	38.46	39.84	44.00	33.06	1.340	0.62	0.27
24 h	38.37	39.20	40.11	42.14	38.19	38.69	1.394	0.98	0.55
CIE value, a*									
0 h	2.78	2.97	2.73	2.40	2.33	2.21	0.178	0.20	0.84
3 h	4.71	4.30	4.03	3.35	4.14	3.96	0.255	0.39	0.38
6 h	4.01	3.93	3.32	3.79	3.47	4.27	0.144	0.89	0.10
9 h	3.12	3.55	3.11	3.37	3.16	3.43	0.134	0.82	0.96
12 h	3.95	3.94	3.52	3.48	3.74	3.90	0.163	0.81	0.39
24 h	4.29	4.83	3.66	4.03	5.11	4.00	0.147	0.71	0.74
CIE value, b*									
0 h	9.00	8.67	6.78	7.58	8.19	7.97	0.383	0.48	0.25
3 h	9.04	9.19	8.75	8.23	7.56	9.59	0.277	0.45	0.14
6 h	9.36	8.97	8.85	8.32	7.96	9.41	0.243	0.52	0.15
9 h	9.45	9.95	8.97	9.11	8.82	9.66	0.245	0.68	0.45
12 h	9.91	9.55	7.75	9.68	9.48	8.74	0.299	0.50	0.49
24 h	12.60	9.50	9.29	9.20	10.01	9.42	0.215	0.68	0.43

SEM, standard error of the mean; CIE, Commission Internationale de l’Eclairage.

1) A, 14% to 11%; B, 15% to 12%; C, 16% to 13%; D, 17% to 14%; E, 18% to 15%; F, 19% to 16%.

(Reduced CP level by 1% for every phase in each treatment group throughout all 4 phases)

## References

[1] Houmard NM, Mainville JL, Bonin CP, Huang S, Luethy MH, Malvar TM (2007). High lysine corn generated by endosperm-specific suppression of lysine catabolism using RNAi. Plant Biotechnol J.

[2] Chen HY, Yi XW, Zhang GJ (2011). Studies on reducing nitrogen excretion: I. net energy requirement of finishing pigs maximizing performance and carcass quality fed low crude protein diets supplemented with crystalline amino acids. J Anim Sci Biotechnol.

[3] Zhao Y, Tian G, Chen D (2019). Effect of different dietary protein levels and amino acids supplementation patterns on growth performance, carcass characteristics and nitrogen excretion in growing-finishing pigs. J Anim Sci Biotechnol.

[4] Yue LY, Qiao SY (2008). Effects of low-protein diets supplemented with crystalline amino acids on performance and intestinal development in piglets over the first 2 weeks after weaning. Livest Sci.

[5] Li N, Xie CY, Zeng XF, Wang DH, Qiao SY (2018). Effects of dietary crude protein level and amino acid balance on growth performance, carcass traits and meat quality of finishing pigs. J Anim Nutr.

[6] Committee on Nutrient Requirements of Swine, National Research Council (1998). Nutrient requirements of swine.

[7] Prandini A, Sigolo S, Morlacchini M, Grilli E, Fiorentini L (2013). Microencapsulated lysine and low-protein diets: effects on performance, carcass characteristics and nitrogen excretion in heavy growing-finishing pigs. J Anim Sci.

[8] Kerr BJ, Mckeith FK, Easter RA (1995). Effect on performance and carcass characteristics of nursery to finisher pigs fed reduced crude protein, amino acid-supplemented diets. J Anim Sci.

[9] Qin CF, Huang P, Qiu K (2015). Influences of dietary protein sources and crude protein levels on intracellular free amino acid profile in the longissimus dorsi muscle of finishing gilts. J Anim Sci Biotechnol.

[10] Yi XW, Zhang SR, Yang Q, Yin HH, Qiao SY (2010). Influence of dietary net energy content on performance of growing pigs fed low crude protein diets supplemented with crystalline amino acids. J Swine Health Prod.

[11] Figueroa JL, Lewis AJ, Miller PS, Fischer RL, Gómez RS, Diedrichsen RM (2002). Nitrogen metabolism and growth performance of gilts fed standard corn-soybean meal diets or low-crude protein, amino acid-supplemented diets. J Anim Sci.

[12] Tuitoek K, Young LG, De Lange CF, Kerr BJ (1997). The effect of reducing excess dietary amino acids on growing-finishing pig performance: an elevation of the ideal protein concept. J Anim Sci.

[13] Jørgensen H, Jakobsen K, Eggum BO (1992). The influence of different protein, fat and mineral levels on the digestibility of fat and fatty acids measured at the terminal ileum and in faeces of growing pigs. Acta Agric Scand A Anim Sci.

[14] Adams KL, Jensen AH (1985). Effect of dietary protein and fat levels on the utilization of the fat in sunflower seeds by the young pig. Anim Feed Sci Technol.

[15] Riedel SM, Field WE (2013). Summation of the frequency, severity, and primary causative factors associated with injuries and fatalities involving confined spaces in agriculture. J Agric Saf Health.

[16] Liu S, Ni JQ, Radcliffle JS, Vonderohe C (2017). Hydrogen sulfide emissions from a swine building affected by dietary crude protein. J Environ Manage.

[17] Committee on Nutrient Requirements of Swine, National Research Council (2012). Nutrient requirements of swine.

[18] Latimer GW, AOAC International (2012). Official methods of analysis of AOAC International.

[19] Roux ML, Donsbough AL, Waguespack AM (2011). Maximizing the use of supplemental amino acids in corn-soybean meal diets for 20- to 45-kilogram pigs. J Anim Sci.

[20] Bergner H, Adler-Nissen J, Eggum BO, Munck L (1977). Protein evaluation and protein metabolism. Biochemical aspects of new protein food. FEBS 11th Meeting.

[21] Coma J, Zimmerman DR, Carrion D (1996). Lysine requirement of the lactating sow determined by using plasma urea nitrogen as a rapid response criterion. J Anim Sci.

[22] Chen HY, Miller PS, Lewis AJ, Wolverton CK, Stroup WW (1995). Changes in plasma urea concentration can be used to determine protein requirements of two populations of pigs with different protein accretion rates. J Anim Sci.

[23] Han IK, Kim JH, Chu KS, Xuan ZH, Sohn KS, Kim MK (1998). Effect of phase feeding on the growth performance and nutrient utilization in finishing pigs. Asian-Australas J Anim Sci.

[24] Bergsjo B, Langseth W, Nafstad I, Jansen JH, Larsen HJ (1993). The effects of naturally deoxynivalenol-contaminated oats on the clinical condition, blood parameters, performance and carcass composition of growing pigs. Vet Res Commun.

[25] Matthews JO, Gentry LR, Chapa AM (1998). Changes in plasma metabolites and hormones in pigs relative to time of feeding. J Anim Sci.

[26] Van der Schoor SRD, Goudoever JBV, Stoll B (2001). The pattern of intestinal substrate oxidation is altered by protein restriction in pigs. Gastroenterol.

[27] Baxmann AC, Ahmed MS, Marques NC (2008). Influence of muscle mass and physical activity on serum and urinary creatinine and serum cystatin C. Clin J Am Soc Nephrol.

[28] O’connell JM, Callan JJ, O’doherty JV (2006). The effect of dietary crude protein level, cereal type and exogenous enzyme supplementation on nutrient digestibility, nitrogen excretion, faecal volatile fatty acid concentration and ammonia emissions from pigs. Anim Feed Sci Technol.

[29] Zervas S, Zijlstra RT (2002). Effects of dietary protein and oathull fiber on nitrogen excretion patterns and postprandial plasma urea profiles in grower pigs. J Anim Sci.

[30] Wang YM, Yu HT, Zhou JY (2019). Effects of feeding growing-finishing pigs with low crude protein diets on growth performance, carcass characteristics, meat quality and nutrient digestibility in different areas of China. Anim Feed Sci Technol.

[31] Wang Y, Zhou J, Wang G, Cai S, Zeng X, Qiao S (2018). Advances in low-protein diets for swine. J Anim Sci Biotechnol.

[32] Le PD, Aarnink AJA, Jongbloed AW, Vander Peet-Schwering CMC, Ogink NWM, Verstegen MWA (2007). Effects of dietary crude protein level on odour from pig manure. Animal.

[33] Hayes ET, Leek ABG, Curran TP (2004). The influence of diet crude protein level on odour and ammonia emissions from finishing pig houses. Bioresour Technol.

[34] Powers WJ, Zamzow SB, Kerr BJ (2007). Reduced crude protein effects on aerial emissions from swine. Appl Eng Agric.

[35] Liu S, Ni JQ, Radcliffe JS, Vonderohe CE (2017). Mitigation of ammonia emissions from pig production using reduced dietary crude protein with amino acid supplementation. Bioresour Technol.

[36] Stewart LL, Kil DY, Ji F (2013). Effects of dietary soybean hulls and wheat middlings on body composition, nutrient and energy retention, and the net energy of diets and ingredients fed to growing and finishing pigs. J Anim Sci.

[37] Fang LH, Jin YH, Do SH (2019). Effects of dietary energy and crude protein levels on growth performance, blood profiles, and carcass traits in growing-finishing pigs. J Anim Sci Technol.

[38] Hong JS, Lee GI, Jin XH, Kim YY (2016). Effect of dietary energy levels and phase feeding by protein levels on growth performance, blood profiles and carcass characteristics in growing-finishing pigs. J Anim Sci Technol.

[39] Zhu YP, Zhou P, Li JL, Zhang L, Gao F, Zhou GH (2017). Effects of adding cysteamine to low-protein amino acid balanced diet on growing pork quality and related gene expression. J Anim Husb Vet Med.

[40] Li N, Xie CY, Zeng XF, Wang DH, Qiao SY (2018). Effects of dietary crude protein level and amino acid balance on growth performance, carcass traits and meat quality of finishing pigs. J Anim Nutr.

